# Polyploid lineages in the genus *Porphyra*

**DOI:** 10.1038/s41598-018-26796-5

**Published:** 2018-06-06

**Authors:** Elena Varela-Álvarez, João Loureiro, Cristina Paulino, Ester A. Serrão

**Affiliations:** 1 CCMAR Centro de Ciências do Mar, CIMAR Laboratório Associado, Universidade do Algarve, Campus de Gambelas, 8005-139 Faro, Portugal; 2 Centre for Functional Ecology, Department of Life Sciences, University of Coimbra, Calçada Martim de Freitas, 3000-465 Coimbra, Portugal

## Abstract

Whole genome duplication is now accepted as an important evolutionary force, but the genetic factors and the life history implications affecting the existence and abundance of polyploid lineages within species are still poorly known. Polyploidy has been mainly studied in plant model species in which the sporophyte is the dominant phase in their life history. In this study, we address such questions in a novel system (*Porphyra*, red algae) where the gametophyte is the dominant phase in the life history. Three *Porphyra* species (*P*. *dioica*, *P*. *umbilicalis*, and *P*. *linearis*) were used in comparisons of ploidy levels, genome sizes and genetic differentiation using flow cytometry and 11 microsatellite markers among putative polyploid lineages. Multiple ploidy levels and genome sizes were found in *Porphyra* species, representing different cell lines and comprising several cytotype combinations among the same and different individuals. In *P*. *linearis*, genetic differentiation was found among three polyploid lineages: triploid, tetraploid and mixoploids, representing different evolutionary units. We conclude that the gametophytic phase (*n*) in *Porphyra* species is not haploid, contradicting previous theories. New hypotheses for the life histories of *Porphyra* species are discussed.

## Introduction

Polyploidy, the increase in genome size by the acquisition of more than one set of chromosomes has been a key factor in eukaryote evolution. In fact, most flowering plants and vertebrates descend from polyploid ancestors^1^. In angiosperms, many species have been suggested to have polyploid ancestry^2^. While whole genome duplication (WGD) is now accepted as an important evolutionary force, the genetic factors and the life history implications affecting the existence and abundance of polyploid lineages are still poorly understood^3–5^. Recent studies have also demonstrated that polyploid genomes can be highly dynamic and undergo rapid structural and functional changes. These findings have renewed the interest in examining WGD as an evolutionary process^6,7^, which coupled with molecular genetics and phylogenetic analyses can provide new insights into the evolution of the genome^8^.

In polyploid research, most of the systems studied are higher plants (e.g. *Brassica*, Cotton, Maize, *Spartina*, Wheat, *Tragopogon*) representing life cycles in which the sporophyte is the dominant phase in a complex cycle. This cycle alternates between sporophytes (spore-producing organisms) and gametophytes (gamete-producing organisms)^9^. Although the sporophyte generation dominates in most extant lineages of land plants, in the earliest groups of land plants and also in several macroalgal groups, the gametophyte generation was (and it still is) the dominant component of the life cycle. Up to date, little is known about polyploidy in system groups that have the gametophyte as the dominant phase. This study uses red algae, an Archaeplastida group on the evolutionary line leading to plants, to study polyploid processes in a “gametophyte dominant” system. By studying polyploidy in such a life cycle, it may be possible to gain insights into polyploid evolution and to address questions related to the origins of polyploid lineages/individuals, the genetic consequences of genome duplication, and/or the importance of the triploid bridge in the origin of tetraploid populations, a topic still under debate^10–12^.

*Porphyra* is a polyphyletic genus of red algae with approximately 57 recognized species^13^ with similar morphology and with a wide variety of life history strategies^14^. A recent taxonomic revision of the *Bangiales* created new genera, and many species previously classified as *Porphyra* species have been transferred to other genera within the *Bangiales*, such as *Pyropia* and *Wildemania* among others^15^. Although this and other molecular studies (e.g. refs^16,17^) have revealed more species diversity in *Porphyra* than was previously understood, little is known about the mechanisms of speciation in the genus *Porphyra*. Three of the recognized *Porphyra* species are the subject of this study: *Porphyra dioica* J Brodie & L. M. Irvine, *Porphyra linearis* Greville, *and Porphyra umbilicalis* Kützing. It is worth to note that although the gametophyte is the dominant phase in their life history, each species has a different mating system in Southern Europe: *Porphyra dioica* has mostly individuals with separate sexes (dioecious), *P*. *umbilicalis* can be hermaphroditic with both sexes in the same individual and gametangia separated in different sections of the thallus (monoecious) or also dioecious, and *P*. *linearis* has individuals with both systems, dioecious and hermaphroditic, but probably as a consequence of a protandrous system (or sequential hermaphroditism, with males being formed first, later becoming females).

*Porphyra* species typically have a heteromorphic life cycle consisting of a gametophytic blade phase (haploid) and a filamentous sporophytic phase called the conchocelis (diploid). The conchocelis phase of *Porphyra* species is difficult to find in nature^18^ with only a few *in situ* records (e.g. refs^19,20^), in contrast with the gametophytic blades, which are common and abundant in nature. The first description of the *Porphyra* life cycle by^21^ revolutionized the farming of this edible seaweed. Drew proved that *Conchocelis rosea* Batters, originally described as a separate species, was the germinated carpospores (sporophyte, phase 2*n*) from the *Porphyra* blades (gametophyte, phase *n*). It has been assumed since then than two ploidy levels are involved in the *Porphyra* life cycles and several chromosome studies have corroborated the ploidy level of both phases, with the gametophytic blade being haploid and the sporophytic carpospores/conchocelis being diploid (e.g. refs ^22–25^). Drew’s study enabled mass cultivation and the development of the aquaculture industry of *Porphyra* (Nori, laverbread), the single most valuable marine product in the orient with a current retail value over $1.3 billion per year^26^.

Recently, within the context of the project NORIGENOMICS (PTDC/MAR/099698/2008), microsatellite markers have been developed for the three *Porphyra* species that are the subject of this study, to describe the population structure and historical biogeography of these species in the North Atlantic. These markers have allowed us to detect gametophytic blades of *Porphyra* possessing heterozygous genotypes with 1–6 alleles per individual in many samples along European coasts (Varela-Álvarez *et al*., unpublished). These findings led us to question if different ploidy levels exist on different individuals/strains and or populations and whether these may be the result of hybridization with other *Porphyra* species/lineages. However, if polyploids exist in our populations/species, how these results can be integrated into the haploid/diploid life history assumed by^21^ is an enigma to be resolved. The origins of polyploid lineages, strains, or individuals within the species-complex, have yet to be determined.

The main objective of this study was to assess the existence of putative polyploid lineages in species of *Porphyra* with different mating systems and, if present, to get insights into the mode of origin of polyploids (i.e., allopolyploidy and/or autopolyploidy). A second objective was to use the evidence from ploidy levels together with the estimation of genetic differentiation among the ploidy types found to understand if the species/cytotypes maintain gene flow. Finally, we expected that these results would provide insight into the evolutionary consequences of ploidy variation for the life history of species of *Porphyra* representing different mating systems; and to contrast hypothetical models for the evolution of polyploidy in organisms where the gametophyte is the dominant phase, relative to those where it is the sporophyte. Besides improving the understanding of the evolutionary dynamics in polyploid complexes in non-specialized plant species, our study provides evidence of a new evolutionary mechanism in the economically important genus *Porphyra* and that might perhaps be common in other red algae.

## Material and Methods

### Sampling collection

A total of 122 blades (*P*. *dioica*, 40 blades; *P*. *umbilicalis*, 35 blades; and *P*. *linearis*, 47 blades) were collected in order to assess their ploidy levels, genome sizes and genetic differentiation among putative polyploid lineages. The localities visited were: Buarcos, Figueira da Foz (40°10′N, 8°53′W) (for *P*. *dioica* and *P*. *umbilicalis*) and Belém, Lisboa (38°41′N, 9°12′W) (for *P*. *linearis*), both in Portugal. Each individual was examined in the binocular microscope and up to 4 sections were made per blade in duplicate (one replicate for genetic analyses and a second replicate for flow cytometric analyses) for further analyses: vegetative blade, zygotospores, female gametes and spermatia (male gametes). The terms zygotosporangia and zygotospores are used to replace carposporangia and carpospores as proposed by Guiry^27^ for the *Bangiales*.

### Flow cytometry

The nuclear DNA content of *Porphyra* spp. was estimated by flow cytometry using fresh material. Nuclei were released following the procedure of Loureiro *et al*.^28^ by chopping approximately 1 cm^2^ of each blade section with a razor blade together with 50 mg of fresh leaf tissue *Solanum lycopersicum* (internal reference standard with 2 C = 1.96 pg^29^) in 1 mL of WPB buffer^28^ to ensure that nuclei of both species were exposed to identical chemical and mechanical conditions. To double check for the existence of smaller genome sizes we also applied to a small set of samples a standard of smaller genome size, *Raphanu*s *sativus* (2 C = 1.11 pg)^29^. The nuclear suspension was then filtered through a 30 μm nylon filter to remove large fragments, and 50 μg/mL of PI - Propidium iodide (Fluka, Buchs, Switzerland) together with 50 μg/mL of RNase (Fluka, Buchs, Switzerland) were subsequently added to respectively stain the nuclei and prevent staining of double-stranded RNA. After incubation for 5 min, the fluorescence intensity of an average of 2,000 nuclei per sample was analysed using a Partec CyFlow Space flow cytometer (Partec GmbH, Görlitz, Germany), equipped with a green solid state laser for PI excitation. The G_1_ peak of the standard was set to channel 720 and the amplification system settings were then kept at a constant voltage and gain throughout the experiment. For the data analyses, measurements were acquired using FloMax software v2.4d (Partec GmbH) in the form of six graphs: fluorescence pulse integral in linear scale (FL) (Graph 1); FL vs. time (Graph 2); side light scatter (SSC) vs. forward light scatter (FSC), both in logarithmic (log) scale (Graph 3); FL vs. fluorescence pulse height (Graph 4); FL vs. FSC (Graph 5) and FL vs. SSC (Graph 6). A region of interest comprising mostly the isolated nuclei was defined in the FL vs. SSC cytogram and subsequently used to gate all the other graphs. The presence of aggregates (doublets or triplets) was evaluated in the FL vs. fluorescence pulse height cytogram. The genome size (in picograms, pg) of each sample was determined according to the following formula: (mean PI fluorescence of the sample/mean PI fluorescence of the standard nuclei) * nuclear DNA content of the standard nuclei. The reliability of the genome size measurements was verified by evaluating the quality of the flow cytometry histograms based on the coefficient of variation (CV) of the G_1_ peaks and the background debris, and the CV of the genome size estimation of each isolate based on three independent measurements.

### DNA extraction, PCR and genotyping

For genotyping, the same individuals (gametophytic blades) used previously for flow cytometry were scored and DNA extractions were obtained from up to 4 sections of each blade referred above.

Genomic DNA was isolated using the LiCl extraction protocol described by^30^ as modified by^31^. Genotyping using 11 selected microsatellite markers^32,33^ generated 121 multilocus genotypes (see Table S1 for sources, loci and amplification details). Polymerase chain reactions (PCR) for all markers were performed separately in a 20 μl reaction volume containing 5–50 ng genomic DNA. Amplifications using an Applied Biosystems thermal cycler (GeneAmp 2720) were conducted by the PCR programs and conditions described in^32,33^. Amplified fragments were separated electrophoretically using an ABI PRISM 3130xl (Applied Biosystems) automated capillary sequencer at CCMAR, Portugal, and sized with GeneScan-350ROX size standard.

### Genetic analyses

Calculation of genetic distances for all pairwise comparisons and estimation of genetic differentiation were performed on the whole data set and in 19 groups according to the ploidy levels found (see results below). Ploidy levels in genotypes were determined by flow cytometry and also by the maximum number of alleles found in a genotype for comparison. Number of alleles, allele range, and unbiased total heterozygosity were determined per group containing similar genotypes. The indices of heterozygosity are used as a theoretical index of genetic diversity; they do not represent observed heterozygosity. For mixoploids (blades containing more than one ploidy level) and samples with different types of cells mixed (e.g. zygotospores mixed with vegetative cells), the highest ploidy level of the cell lines found in the sample was considered. For multiploid gametes (gametes of several ploidy levels produced in the same blade, see results), the basic ploidy level found in the samples was considered (3*x* or 4*x*). Alleles were scored in GENEMAPPER v.4.1 (Applied Biosystems). Binning and allele rounding performed by GENEMAPPER v.4.1 was also checked with TANDEM^34^. The calculation of genetic diversity among the samples was estimated using GENODIVE, which allows analysis of polyploid data^35^. Summary statistics of genetic diversity within populations were calculated, including N: Number of alleles (total number of alleles found), Eff_num: Effective number of alleles (the number of equally frequent alleles it would take to achieve a given level of gene diversity), Hs: Heterozygosity within populations (the expected frequency of heterozygotes within populations, assuming Hardy-Weinberg equilibrium), also known as Gene Diversity, and Ht: Total heterozygosity, the expected frequency of heterozygotes over all populations, assuming Hardy-Weinberg equilibrium. Both Hs and Ht have a correction for sampling bias stemming from sampling a limited number of individuals per population. Each parameter was calculated with and without the maximum likelihood-correction for allele dosage in polyploids implemented in GENODIVE^35^.

#### Genetic distances and genotypic differentiation

Genetic distance represents the amount of between-population difference in allele frequency summed over all loci. Genetic differentiation between individual loci was measured by pairwise genetic distances (Nei G_ST_/JostD) for all pairs of groups between the three species and among groups within each species (with and without the maximum likelihood-correction for allele dosage for polyploids) and they were also calculated with GENODIVE^35^.

In order to illustrate the variation in genetic diversity and genotypic differentiation (distribution of genotypes among the populations), a principal component analysis (PCA) based on allelic variation across all genotyped individuals was performed using GENODIVE^35^. Genetic population structuring among the three species and among the ploidy levels found was evaluated with the software STRUCTURE^36^. Structure analysis applies a Bayesian clustering approach and it was used to identify the population structure inferred from microsatellites. Structure analyses were performed with all individuals combined into one dataset for a global analysis and also using one data set per species. Analyses were run without any *a priori* population assignments and admixture was allowed. We assumed a model where the number of clusters (*K*) was unknown and the population structure was inferred (lower and upper bound for *K* equal to 1 and 20, respectively). The modal value of ΔK distribution for the posterior probability of the data for a given *K* was used as an indicator of the strength of the signal detected by Structure and considered as the real number of *K* clusters^37^. Each *K* was replicated 10 times with 10,000 Markov Chain Monte Carlo (MCMC) iterations after a burn-in period of 50,000, without any prior information on the ploidy and/or species group of each sampled individual. The average membership coefficients for the 10 simulation runs of a given *K* value were generated by CLUMPP v1.1.2^38^ and a graphical presentation of the average membership coefficients for each isolate was generated in Microsoft Excel. An estimate of the true number of populations, *K*, was calculated using an *ad hoc* statistic-based approach implemented in STRUCTURE HARVESTER v0.6.1^39^, as described previously. To avoid bias caused by missing values in the PCA and Structure analyses, missing data were replaced with random values randomly picked from the allele pool of each population as in^40^. Finally, in order to evaluate the degree of association between genotypic variation and ploidy levels, a neighbour-joining (NJ) network was generated from a matrix of pairwise^41^ genetic distances of individuals (genotypes), using POPULATIONS 1.2^42^.

### Data availability

All the data (flow cytometry data set and genotypes) are available in supplementary information online (Appendices 1 and 2).

## Results

### Results from the cytometric approach

**Figure 1:**
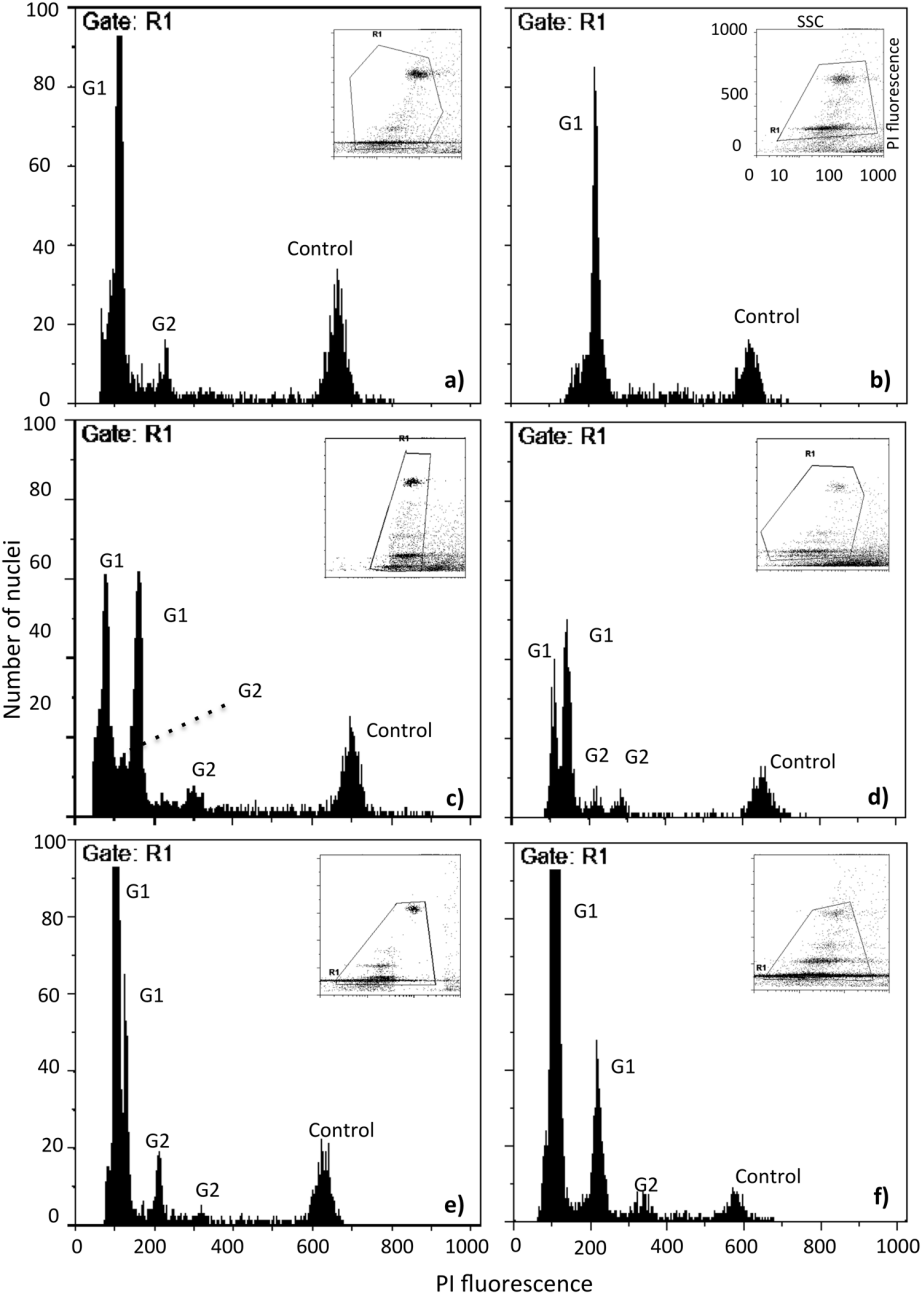
Histograms of relative fluorescence intensities in *Porphyra*. Examples of histograms of relative fluorescence intensities obtained through simultaneous flow cytometric analysis of propidium iodide-stained nuclei of *Porphyra* (peaks 1–4) and internal reference standard *S*. *lycopersicum* (peak at the right; with 2C = 1.96 pg). X-axis: Fluorescence (channel number). Y-axis: nuclei counts in each sample. (**a**) Triploid gametes in *P*. *dioica*. (**b**) Octoploid zygotospores from a tetraploid cytotype in *P*. *umbilicalis*. (**c**) Mixoploid cytotype 2*x*/4*x* in *P*. *linearis*. (**d**) Mixoploid cytotype 3*x*/4*x* in *P*. *linearis*. (**e**) Male gametes 3*x* and 4*x*. (**f**) Multiploid gametes 4*x* in *P*. *dioica*. Insets show cytograms of side scatters in logarithmic scale (sslog, axis x) vs. Propidium iodide fluorescence (PI, axis y) of nuclear suspensions of the same samples.

In total, 217 flow cytometric analyses were performed (*P*. *linearis*: 71, *P*. *umbilicalis*: 69, *P*. *dioica*: 77) using 122 blades (Appendix 1). Genome sizes in this study varied from 0.2 to 1.6 pg (Table 1 and Appendix 1). In each individual, 2 to 4 populations of sample nuclei were found (Fig. 1). In most, a minor subpopulation was evident at a fluorescence intensity corresponding to the G_2_-phase. The minimum ploidy level was set in the nuclei population with the lowest genome size, which was found in the gametes (DNA corresponding to the non-replicated haploid chromosome complement). Considering this reference, the remainder genome size values obtained were assigned a ploidy level. Evidence from genotyping (next section) also indicated that gametophytes and gametes presented 2 or more alleles per locus, so being ruled out as haploid; thus, we assigned the gametes with the smaller genome size (0.2 pg) the first ploidy level as 2*x*. Eight different genome size values were obtained across the *Porphyra* samples, whose ratios could only correspond to seven ploidy levels shown in Table 1. The ploidy levels 2*x*, 3*x*, 4*x*, 6*x* and 8*x* were the most common among the samples. Two cell lines were associated with these ploidy levels: cell line 1: 2*x*–4*x*–8*x* and cell line 2: 3*x*–6*x*. In some samples of male gametes, two further genome sizes were obtained, 0.9 pg and 1.2 pg, which correspond with ploidy levels 9*x* and 12*x*. Genome sizes of 1.6 pg/1C corresponded to the G_2_-phase of ploidy levels 8*x* (Table 1).

**Table 1:** Genome size variation and ploidy levels in *Porphyra* species analysed in this study.

Main genome sizes found						Occasional genome sizes found		
Genome size (pg) approx	**0**.**2**	**0**.**3**	**0**.**4**	**0**.**6**	**0**.**8**	0.9	1.2	1.6
Ploidy level	**2** ***x***	**3** ***x***	**4** ***x***	**6** ***x***	**8** ***x***	9*x*	12*x*	G_2_ of 8*x*
*P*. *umbilicalis*	**0**.**19 (0**.**02)**	**0**.**33 (0**.**01)**	**0**.**39 (0**.**06)**	**0**.**67 (0**.**03)**	**0**.**75 (0**.**04)**	1.01 (0.01)	1.20	1.62
*P*. *dioica*	**0**.**19 (0**.**01)**	**0**.**32 (0**.**01)**	**0**.**38 (0**.**02)**	**0**.**68 (0**.**02)**	**0**.**74 (0**.**02)**	1.01 (0.02)	1.13 (0.01)	1.44 (0.02)
*P*. *linearis*	**0**.**19 (0**.**04)**	**0**.**31 (0**.**02)**	**0**.**44 (0**.**06)**	**0**.**64 (0**.**04)**	**0**.**83 (0**.**03)**			

Mean and standard deviation of genome size and ploidy levels in the three *Porphyra* species. Full data set for all the samples is provided in Appendix 1.

**Figure 2:**
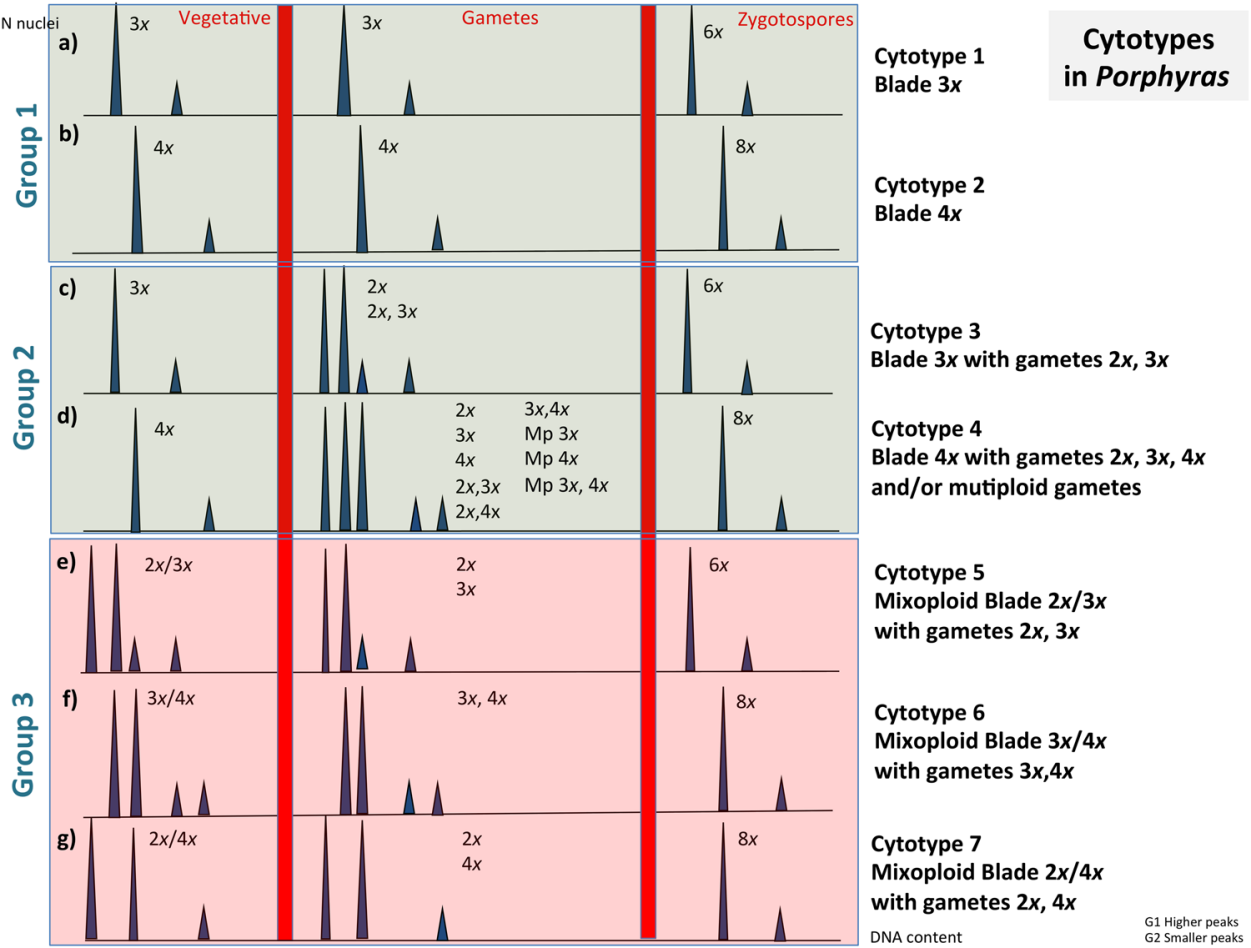
Cytotypes of three *Porphyra* species analysed in this study. Diagram of the correspondence between histograms of DNA content and ploidy levels, in vegetative blades, gametes and zygotospores.

In total, 7 different cytotype combinations were found in these *Porphyra* species that can be classified in three groups (Table 2 and Fig. 2). Group 1 in which gametophytes of each polyploid race (triploid or tetraploid) produced gametes of the same ploidy: (a) Triploid blades 3*x* with 3*x* gametes, (b) Tetraploid blades 4*x* with 4*x* gametes; Group 2 in which gametophytes of each polyploid race (triploid or tetraploid) produced gametes of the same and/or different ploidy, (c) Triploid blades 3*x* with gametes 2*x* and/or 3*x*, (d) Tetraploid blades 4*x* with gametes of the same or different ploidy (2*x*, 3*x*, and/or 4*x*); and Group 3 in which gametophytes belong to mixoploid races in which cell lines 1 and 2 were present in the same individual and they produce gametes of different ploidies also: (e) Mixoploid blades (2*x*/3*x*), (f) Mixoploid blades (3*x*/4*x*) and (g) Mixoploid blades (2*x*/4*x*). In general, for mixoploid blades or “Mx blades” (containing more than one ploidy level), we indicated each ploidy level separated by a “/” (e.g., 2*x*/3*x*). When gametes with the same and lower ploidies were found in the same blade, we separated the different ploidy levels by a “,” (e.g., 2*x*, 3*x*). G1 and G2 on the top of each peak represent nuclei at G_1_-phase and G_2_-phase.

**Table 2:** Cytotypes presence for each species.

Cytotypes	Group 1 Triploids and tetraploids		Group 2 Triploids and tetraploids with gametes of different ploidy than the blade		Group 3 Mixoploids		
	Cytotype 1	Cytotype 2	Cytotype 3	Cytotype 4	Cytotype 5	Cytotype 6	Cytotype 7
	Triploid (3*x*)	Tetraploid (4*x*)	Triploid (3*x*) with gametes 2*x*, 3*x*	Tetraploid (4*x*) with gametes 2*x*, 3*x*, 4*x*, Mp 3*x*, 4*x*	Mixoploid 2*x*/3*x*	Mixoploid 3*x*/4*x*	Mixoploid 2*x*/4*x*
*P*. *umbilicalis*	++	++	++	++	+	−	−
*P*. *dioica*	+	++	−	++	−	−	−
*P*. *linearis*	++	++	+	−	+	++	+

Group 1: Triploids and Tetraploids. Group 2: Triploids and tetraploids with gametes of different ploidy level than the blade. Group 3: Mixoploids. Presence: (++): abundant; (+): present; (−) absent.

In addition, in Group 2, in the Tetraploid race, (Cytotype 4, Fig. 2 and Table 2) some blades produced gametes of a higher ploidy level than the gametophytic blade. We named these gametes as multiploid gametes or “Mp gametes”. We found Mp gametes 3*x*, (3*x*, 6*x*, 9*x*), and Mp gametes 4*x* (4*x*, 8*x*, 12*x*). The 2*x* ploidy level was only found in gametes and in vegetative parts of mixoploids; no single blade was detected as exclusively diploid (2*x*).

The diplophasic phase 2*n* was found among the samples in zygotospores for all the species and it was always 6*x* (in 3*x* blades) and 8*x* (in 4*x* blades). In mixoploids, the zygotospores were only the double of the highest ploidy level (e.g., in 2*x*/3*x* blades, the zygotospores were 6*x*). Furthermore, zygostospores (Zp) were found in either the marginal cells of the blade or in the vegetative part of the gametophytic blade for each polyploid race. When zygotospores and blades were sometimes found mixed with gametes (unfertilized); we indicated each of the ploidy levels of each cell separated by a “+” (e.g., 4*x* + 8*x*).

Although the overall average genome size did not differ among the three *Porphyra* species, the incidence of each cytotype combination differed among them. In roughly, *P*. *linearis* was the species with the highest number of mixoploids (40%) and blades produced gametes of any ploidy level (2*x*, 3*x*, 4*x*). Also, 17.5% of the blades were 4*x* with 4*x* gametes; 37.5% blades were 3*x* with 3*x* gametes, and a small proportion (5%) of triploids produced male gametes with lower ploidy. In *P*. *umbilicalis*, 40% of the individuals were 4*x* with 4*x* gametes, 17% were 3*x* with 3*x* gametes and 37% were either 4*x* or 3*x* that produced gametes with lower ploidy. Mixoploidy was also present, but in a much lower percentage (6%) than in *P*. *linearis*. For *P*. *dioica*: 42% were 4*x* with gametes 4*x*, 3% were triploid 3*x* with gametes 3*x* and 55% were fronds 4*x* with gametes of different ploidy levels.

### Results from the molecular approach

In total, 121 multi-locus genotypes were found (Appendix 2). These where divided in 19 groups according to the species and ploidy level found in the flow cytometric analyses. The number of genotypes in each group varied from 1 to 23, representing “single ploidy levels” (3*x*, 4*x*, 6*x*, 8*x*), and “combined ploidy levels” (2*x*/3*x*; 2*x*/4*x*; 3*x*/4*x*; 4*x* + 8*x*; 3*x* + 8*x*, among other combinations) (Table 3). For all the species, we divided the data in as many groups as possible to decipher if there was any genetic differentiation among them; for example, 6*x* zygotospores coming from triploid blades or from mixoploid blades were separated in two distinct groups. When there was only one replicate for one type of ploidy level, we grouped the samples in a heterogeneous group (e.g. group 10 in *P*. *umbilicalis*) containing genotypes with different ploidy levels and different type of cells in the same sample.

**Table 3:** Genotypes across *Porphyra* spp.

	*P*. *linearis*		*P*. *umbilicalis*		*P*. *dioica*	
(a)	Triploid genotypes 3*x* (15)		Triploid genotypes 3*x* (10)		Triploid genotypes 3*x* (2)	
	Tetraploid genotypes 4*x* (7)		Tetraploid genotypes 4*x* (23)		Tetraploid genotypes 4*x* (21)	
	Mixoploid genotypes Mx (12)				—	
			Multiploid gametes genotypes Mp (1)		Multiploid gametes genotypes Mp (9)	
	—		Other genotypes (2)			
	Genotypes including Zygotospores Zp (6*x* or 8*x*) and other cells of different ploidies (7)		Genotypes including Zygotospores Zp (6*x* or 8*x*) and other cells of different ploidies (3)		Genotypes including Zygotospores Zp (6*x* or 8*x*) and other cells of different ploidies (9)	
(b)	Group 1	3*x* (15)	Group 8	4*x* (23)	Group 11	4*x* (21)
	Group 2	4*x* (7)	Group 9	3*x* (10)	Group 12	3*x* (2)
	Group 3	Mx 2*x*/3*x* (3), Mx 2*x*/4*x* (1)	Group 10	Mp 3*x* (1)	Group 13	Mp 4*x* (3)
	Group 4	Mx 3*x*/4*x* (8)		Blade 4*x* + Gametes 2*x*, 4*x* (2)	Group 14	Mp 3*x* (5)
	Group 5	Blade 3*x* + Zp 6*x* (1), Zp 6*x* (2)		Mx 2*x*/3*x* + Zp 6*x* (1)	Group 15	Mp 3*x*, 4*x* (1)
	Group 6	Mx 2*x*/3*x* + Zp 6*x* (2)		Gametes 2*x* + Zp 6*x* (1)	Group 16	Zp 8*x* (4)
	Group 7	Mx 2*x*/4*x* + Zp 8*x* (1)		Zp 8*x* (1)	Group 17	Blade 4*x* + Zp 8*x* (3)
		Mx 3*x*/4*x* + Zp 8*x* (1)			Group 18	Gametes 2*x* + Zp 8*x* (1)
					Group 19	Gametes 3*x* + Zp 8*x* (1)

(a) Summary of genotypes vs. ploidy levels found for each species: Triploid (3*x*) and tetraploid (4*x*) genotypes found either in vegetative parts of the blade or gametes, (Mx) mixoploid genotypes found in vegetative parts of the blade, (Mp) multiploid gametes with the same or higher ploidy than the vegetative part of the blade, and (Zp) Zygotospores that could be found alone or with vegetative parts of the blade in mixoploids or regular blades and other cells of different ploidies. (b) In detail, all the genotypes found in this study divided in 19 groups according to the exact ploidy levels found. For *P*. *linearis*, Group 1: Triploids (3*x*), Group 2: tetraploids (4*x*), Group 3: Mixoploids 2*x*/3*x* or 2*x*/4*x*, Group 4: Mixoploids 3*x*/4*x*, Group 5: Zygotospores 6*x*, Group 6: Zygotospores 6*x* found in mixoploids (2*x*/3*x*), Group 7: Zygotospores 8*x* found in mixoploids (2*x*/4*x* or 3*x*/4*x*). For *P*. *umbilicalis*, Group 8: tetraploids (4*x*), Group 9: triploids (3*x*), Group 10: genotypes of several types (multiploid gametes 3*x*, blades 4*x* with gametes 2*x* and 4*x*, mixoploids 2*x*/3*x* with zygotospores 6*x*, zygotospores 6*x* and female gametes 2*x*, and zygotospores 8*x*). For *P*. *dioica*, Group 11: tetraploids 4*x*, Group 12: triploids 3*x*, Group 13: multiploid gametes 4*x*; Group 14: multiploid gametes 3*x*, Group 15: multiploid gametes 3*x* and 4*x*, Group 16: zygotospores 8*x*, Group 17: tetraploids 4*x* with zygotospores 8*x*, Group 18: gametes 2*x* and zygotospores 8*x*, Group 19: gametes 3*x* and zygotospores 8*x*. In each case the number of individuals is given in brackets. In (a) and (b), zygotospores are 2*n*, blades and gametes are n. Mixoploid blades contain more than one ploidy level, separated by a “/” (e.g., 2*x*/3*x*). Gametes with the same and lower ploidies found in the same blade, indicated separated by a “,” (e.g., 2*x*, 3*x*). Gametes with the same and higher ploidy levels (e.g. 3*x*, 6*x*, 12*x*) found in the same blade are labelled by “Mp”. Zygotospores (Zp) (the result of fecundation) and blades are sometimes found mixed with gametes (unfertilized); in these cases ploidy levels of each cell are indicated separated by a “+” (e.g. 4*x* + 8*x*). Numbers in brackets represent number of genotypes for each group.

Genetic diversity, both measured as mean number of alleles per locus and the standardized effective number of alleles, was similar for the three species (Table 4). When comparing groups, no genetic differences were found among individuals that produced gametes with lower or multiple ploidy levels and gametes of the same ploidy (data not shown) for any of the species. After applying the maximum likelihood correction for the unknown polyploid dosage, genetic variability statistics did not vary greatly relative to the full data set. Gene diversity (expressed by Hs = 0.5 or Ht = 0.5, approximately) was similar for the three species and for the polyploid lineages for each species. Genetic differentiation expressed as Nei G_ST_ and Jost D (Table 5) was found among some groups in *P linearis* between triploids vs. tetraploids (G_ST_/Jost D: 0.27/0.25) and triploids vs. mixoploids 3*x*/4*x* (G_ST_/Jost D: 0.17/0.15). The rest of the group comparisons presented values close to zero. In addition, no differentiation was found between any groups in the other two species (data not shown).

**Table 4:** Genetic diversity indices among polyploid lineages in the three *Porphyra* species.

	N	Num	Eff_num	Hs	Ht
***Porphyra linearis***					
Total	41	6.45 (1.27)/6.45 (1.27)	1.82 (0.21)/1.65 (0.18)	0.49 (0.06)/0.43 (0.06)	0.51(0.06)/0.46 (0.06)
N phase	34	6.18 (1.18)/6.18 (1.18)	2.06 (0.22)/1.84 (0.19)	0.52(0.06)/0.45 (0.06)	0.54 (0.06)/0.49 (0.06)
3*x*	15	3.09 (0.73)/3.09 (0.73)	2.00 (0.27)/1.85 (0.24)	0.41 (0.07)/0.38 (0.07)	0.41 (0.07)/0.38 (0.07)
4*x*	7	3.18 (0.44)/3.18 (0.44)	2.29 (0.34)/2.03 (0.29)	0.51 (0.08)/0.45 (0.09)	0.51 (0.08)/0.45 (0.09)
2*x*/3*x*; 2*x*/4*x*	4	2.45 (0.34)/2.45 (0.34)	2.11 (0.27)/1.86 (0.23)	0.55 (0.09)/0.47 (0.08)	0.55 (0.09)/0.47 (0.08)
3*x*/4*x*	8	3.72 (0.54)/3.72 (0.54)	2.60 (0.37)/2.15 (0.31)	0.60 (0.07)/0.50 (0.07)	0.60 (0.07)/0.50 (0.07)
***Porphyra umbilicalis***					
Total	39	6.36 (1.15)/6.36 (1.15)	2.60 (0.41)/2.38 (0.38)	0.55 (0.08)/0.51 (0.08)	0.56 (0.08)/0.51 (0.08)
N phase	33	5.63 (1.05)/5.63 (1.05)	2.61 (0.46)/2.34 (0.39)	0.52 (0.08)/0.47 (0.08)	0.52 (0.08)/0.47 (0.09)
3*x*	10	3.54 (0.59)/3.54 (0.59)	2.51 (0.40)/2.26 (0.34)	0.52 (0.08)/0.47 (0.08)	0.52 (0.08)/0.47 (0.08)
4*x*	23	5.00 (1.13)/5.00 (1.13)	2.82 (0.57)/2.49 (0.47)	0.51 (0.08)/0.46 (0.09)	0.51 (0.08)/0.46 (0.09)
***Porphyra dioica***					
Total	41	6.63 (1.03)/6.63 (1.03)	1.91 (0.29)/1.83 (0.27)	0.58 (0.07)/0.53 (0.08)	0.51 (0.07)/0.47 (0.07)
N phase	33	6.09 (1.14)/6.09 (1.14)	2.05 (0.34)/1.96 (0.33)	0.56 (0.08)/0.52 (0.08)	0.52 (0.07)/0.49 (0.08)
3x*	7	3.63 (0.74)/3.63 (0.74)	2.25 (0.35)/2.02 (0.28)	0.59 (0.08)/0.53 (0.08)	0.56 (0.08)/0.51 (0.08)
4x*	24	5.45 (1.12)/5.45 (1.12)	2.36 (0.45)/2.23 (0.43)	0.52 (0.07)/0.48 (0.08)	0.50 (0.07)/0.46 (0.08)

N, Num, Eff_num, Hs and Ht. For each parameter is given the value (right) and the value with maximum likelihood-correction for polyploidy dosage (left). N: Sample size, Num: Number of alleles, Eff_num: Effective number of alleles (the number of equally frequent alleles it would take to achieve a given level of gene diversity), Hs: Heterozygosity within populations (polyploid lineages) or Gene diversity (the expected frequency of heterozygotes within subpopulations, assuming Hardy-Weinberg equilibrium), and Ht: Total Heterozygosity (The expected frequency of heterozygotes over all populations (polyploid lineages), assuming Hardy-Weinberg equilibrium). In brackets standard deviation is shown. In *P*. *dioica*, (*) means all the cytotypes 3*x* or 4*x* are included in the each group: (4*x* *4*x*, Mp 4*x*, 4*x* + Mp 4*x*; 3*x* *3*x*, Mp 3*x*, 3*x* + Mp 3*x*).

**Table 5:** Pairwise genetic distances among groups in *P*. *linearis*.

Nei Gst/Jost D distances				
	3*x*	4*x*	2*x*/3*x*; 2*x*/4*x*	3*x*/4*x*
3*x*	—	0.251	−0.006	0.159
4*x*	0.272	—	0.056	0.023
2*x*/3*x*; 2*x*/4*x*	−0.004	0.050	—	−0.042
3*x*/4*x*	0.178	0.024	−0.047	—

Nei G_ST_^74^ (above) and Jost´s D^75^ (below), polyploid dosage corrected in both parameters, for triploids, tetraploids and mixoploids.

**Figure 3:**
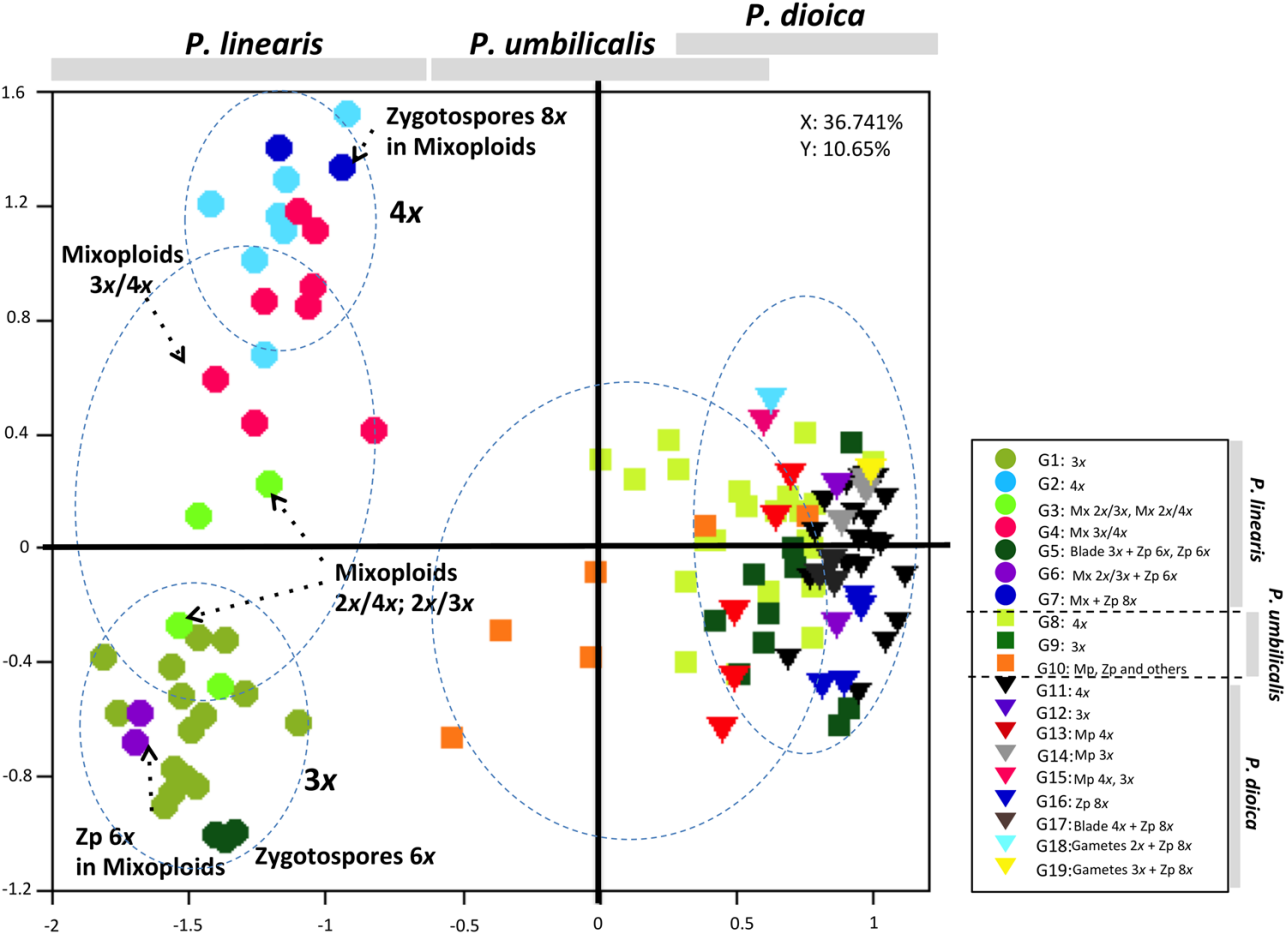
Spatial representation of genetic differentiation within three *Porphyra* species. Principal component analyses (PCA) based on allelic variation at 11 loci. On the right, G1, G2, G3 etc. represent the genotypes in Group 1, Group 2 etc. in Table 3.

**Figure 4:**
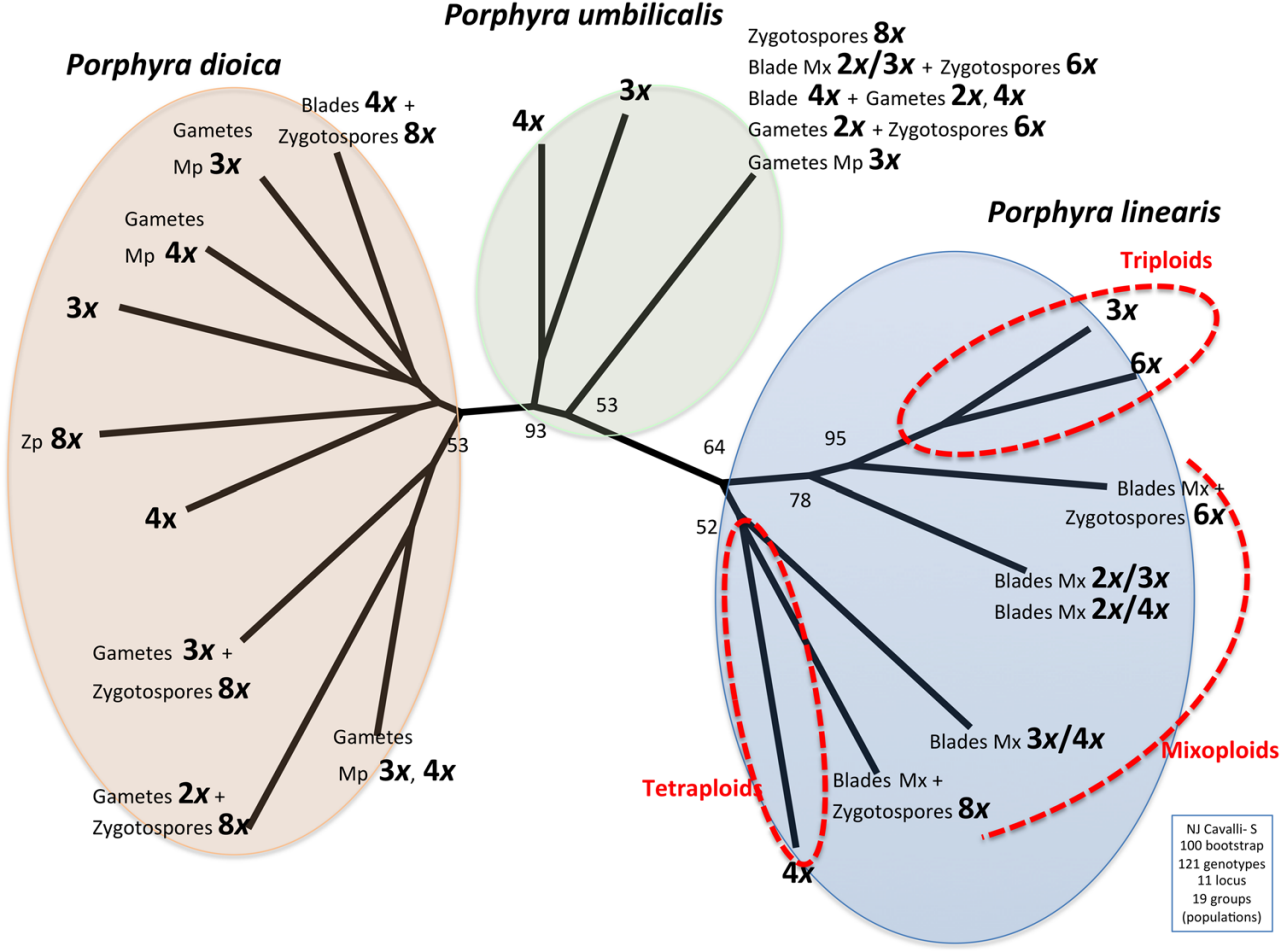
Relationship between genotypic variation among three *Porphyra* species. Neighbour-joining (NJ) network generated from a matrix of pairwise^41^ genetic distances (genotypes), using Populations software. Data from 121 genotypes grouped in 19 groups. The red lines show the well distinct lineages for *P*. *linearis*, triploids, tetraploids (in circles) and the transition mixoploids lineages (dotted line).

**Figure 5:**
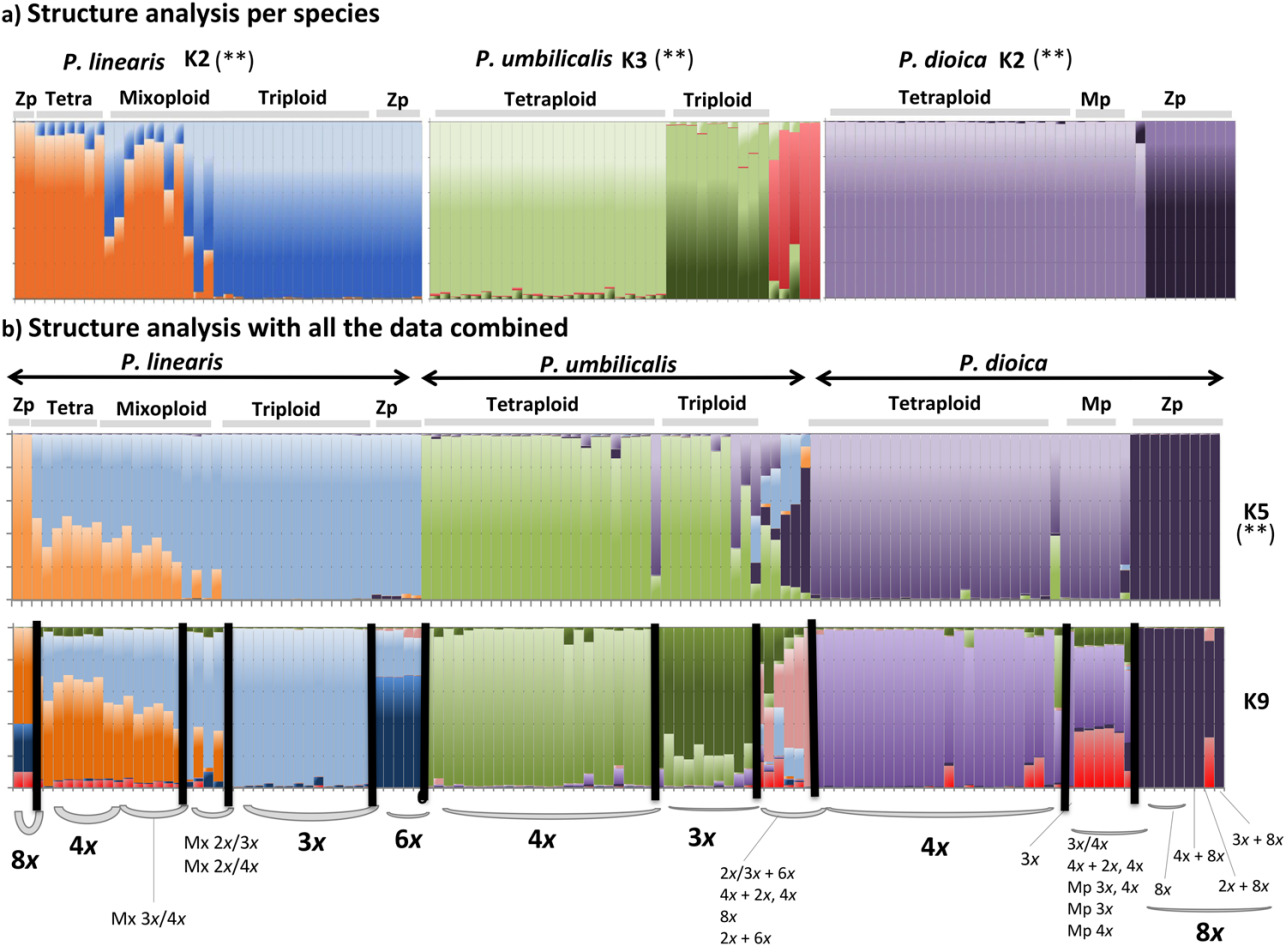
Genetic subdivision of genotypes found in three *Porphyra* species inferred by Structure. Bar colours represent the proportions of individual genotypes attributable to K genetic clusters (K). Ploidy groups per species: Zp = zygotospores, Tetra = tetraploid (genotypes and gametes), Mx = Mixoploid, Mp = multiploid gametes, Triploid (genotypes and gametes). Ploidy types are denoted on the top of the plot, and ploidy levels on the bottom of the plot. Each genotype corresponds to either blades, gametes and/or zygotospores. (**a**) Structure analysis per species separately. (**b**) Structure analysis with all the data combined. Best Ks for each group are highlighted with the symbol (**).

When performing a PCA (principal component analyses) with all the genotypes together, the three species were strongly differentiated (Fig. 3), as were some ploidy groups within one species (*P*. *linearis*), indicating reproductive isolation mediated by ploidy. Within *P*. *linearis*, genotypes with different ploidy levels (triploids vs. tetraploids vs. mixoploids) were genetically differentiated. Also, the genotypes of the 2*n* phase, zygotospores 6*x* and 8*x*, were closely related to cytotypes 3*x* and 4*x* respectively. Within *P*. *umbilicalis* and *P*. *dioica*, no genetic structuring by ploidy level could be identified in the PCA (Fig. 3), but some introgression between these species was suggested. The NJ network (Fig. 4) highlighted clearly the genetic differentiation by species, and within *P*. *linearis* and *P*. *umbilicalis*, also according with ploidy level (triploid vs. tetraploids vs. mixoploids or multiple ploidy levels). However, for *P*. *dioica*, there was not a clear structure among triploids and tetraploids. The STRUCTURE analyses separated well the three species but also showed genetic lineages within each species (Fig. 5) with best *K*s (clusters/groups with similar/related genotypes) found for *P*. *linearis K* = 2 (two clusters), *P*. *umbilicalis K* = 3 (three clusters), and *P*. *dioica K* = 2 (two clusters) (Fig. 5a). With all the data set, the best *K* was *K* = 5 (**Δ**K criterion), results were similar to the analyses of species separately, including distinct clusters for each species, and then distinct genetic lineages for each species. With further subdivision until *K* = 9 all groups different ploidy levels within each species were revealed (see Fig. 5b). Clusters in each analysis are represented by colours, and individuals are represented as columns. Within each column (individual), the extent of the component colours indicates the magnitude of the membership coefficient (Q) corresponding to each cluster. Up to three genetically distinct groups, coincident with triploid, tetraploid and mixoploid/multiploid individuals were identified within both *P*. *dioica* and *P*. *umbilicalis*, either in the analyses per single species or in the analyses with all the data combined. These three main ploidy types in *P*. *linearis* were not coincident with only three genetic groups. These results also indicate some levels of admixture or cryptic presence of common genotypes in *P*. *dioica* and *P*. *umbilicalis*.

## Discussion

### Polyploid races in *Porphyra*

Our results show that the gametophytes of the three *Porphyra* species studied are polyploid. *Porphyra* species showed high variability in genome sizes (eight), ploidy levels (seven), with two different cell lines found and at least three distinct lineages which comprised seven cytotypes. We show that the gametophytic phase (*n*) in *Porphyra* is not haploid, in disagreement with the assumptions derived from Drew^21^ landmark paper. Our results confirm the haplophasic/diplophasic cycle in *Porphyra*, with replicated and unreplicated nuclei, but not with the ploidy levels previously assumed, nor the simplicity of only two ploidy levels (haploid vs. diploid) assumed. The terminology haplophasic/diplophasic phase is already described for other plant species^43^.

There was important genetic differentiation and structure among the three species (Figs 3–5). Within each species, genetic differentiation of the polyploid types was different; in *P*. *linearis*, genetic differentiation between polyploid types (triploids vs. tetraploids vs. mixoploids) was strong; in *P*. *dioica* there was no significant genetic differentiation (most of the genotypes were tetraploid); and in *P*. *umbilicalis*, there was some degree of genetic differentiation (weak) which revealed discrete genetic clusters. Gene flow in polyploid lineages in these *Porphyra* species is likely mediated either with intermediate lineages (mixoploids) or by individuals that release gametes of lower ploidy levels that can fecundate different cytotypes. In *P*. *dioica*, the population is dominated by tetraploid individuals with gametes of multiple ploidy levels.

We also support the idea that the *Porphyra* system is a complex system formed by autopolyploids, mixoploids and allopolyploids that interact among and between them. Triploids and tetraploids are normally originated by the fusion of gametes of different ploidies originated by irregular mitotic processes. Mixoploids are more likely originated by meiotic processes in the germination of the conchospore into four different tetrads (genetic chimeras, see section below) as reported before by previous authors for some *Porphyra* spp. (e.g.ref^44^), and consequently different cell lines within the same individual. We have not tested here the hypothesis of whether gametes from one species fecundate gametes from another species (with the same or different ploidy level), but we have observed some shared genotypes between *P*. *dioica* and *P*. *umbilicalis* (Figs 3–5). Yet, allopolyploids have been already observed by^45^ for some Japanese species in nature.

### Mixoploidy, genetic chimeras and multiploid gametes

Genetic chimeras or organisms with more than one cell line have been reported for several plant species (e.g. ref^46^), including algal species such as *Gracilaria tikvahiae* McLachlan^47,48^ and a *Porphyra* species: *P*. *yezoensis*^44,49,50^. In *Porphyra yezoensis* Ueda (actual name: *Pyropia yezoensis* (Ueda) M.S. Hwang & H.G. Choi) meiosis occurs as the conchospores germinate and the four resultant cells, which all have different genetic compositions, form the mature blade through successive divisions^44^. However, Niwa *et al*.^51,52^, have been reporting both chimeric blades and uniform blades, the last ones associated to asexual reproduction. In the species analysed in the current study, we have detected chimeric blades (mixoploids) and blades with one vegetative cell line (triploids and tetraploids), of which some individuals produced gametes of the same ploidy and others produced gametes of multiple ploidy levels. Three types of mixoploidy have been discovered: 2*x*/3*x*, 2*x*/4*x*, and 3*x*/4*x*. When analysing samples randomly collected in each blade, we always obtained the same result. Thus, it can be concluded that the formation of mixoploids for the samples examined was not sectorial, and the two different cell lines seem to be mixed within the blade. However, further research would be needed to study and confirm this aspect in detail.

We hypothesize that in our polyploid system, mixoploids or blades with two cell lines are a bridge between ploidy types. In polyploid research, triploids are believed to act as bridges between ploidy types; in such a role, they mediate the formation of tetraploids from diploids or facilitate gene flow between diploids and tetraploids^4^. Triploidy is associated to a long-term strategy, that could produce either asexual progeny or sexual progeny. For example, with a specialized meiosis, triploids can produce haploid sperm and diploid eggs (e.g. in frogs)^53^, or diploid sperm and haploid eggs (e.g. in plants)^54,55^. The mixoploids blades found here could reflect a long-term strategy in the *Porphyra* system of concealing and admixture of different cytotypes in the same species, preventing the exclusion of new cytotypes after emergence (similarly as the minority cytotype exclusion theory^56^). In the same way, blades that produce gametes of different ploidy levels act as bridges among cytotypes. The generation of gametes of lower ploidy (2*x*, 3*x*) in tetraploid blades prevents the elimination of triploids and also ensures the admixture of different polyploid lineages.

We can’t answer how the production of gametes of different ploidy levels is performed. The processes involved in the DNA reduction in *Porphyra* are yet to be described. Decrease in genome size has been reported to be adaptive. Rapid elimination of DNA sequences from autopolyploid genomes seems to occur in species such as *Elymus elongatus*, where both newly synthesized autopolyploids as well as natural accessions showed a loss of 10% DNA compared with their diploid progenitors^57^. However, this reduction is performed in a different way, since genome reduction is done in all the cells in the plant, not only the reproductive cells.

In the case of gametes of higher ploidies (“multiploid gametes”) found in *P*. *dioica* (9*x*, 12*x*) the only explanation to these sizes is the presence of spontaneous chromosome duplication/triplication phenomena. We discard the hypothesis of the presence of aggregates (doublets or triplets) since no changes in nuclei pulse width were detected among the nuclear populations, as expected if doublets or triplets existed (doublets show larger pulse width when compared with singlets). However, spontaneous chromosome duplication has already been reported in *Porphyra* species^58^ in^59^. However, as no blades of higher ploidy levels (>4*x*) were found, we presumed that those gametes are unviable. A simple explanation for this would be that since only male gametes presented those ploidy levels, fecundation with female gametes is not possible. Still, further research is needed to clarify this point.

### Proposed life histories for *Porphyras* in the Iberian Peninsula

**Figure 6:**
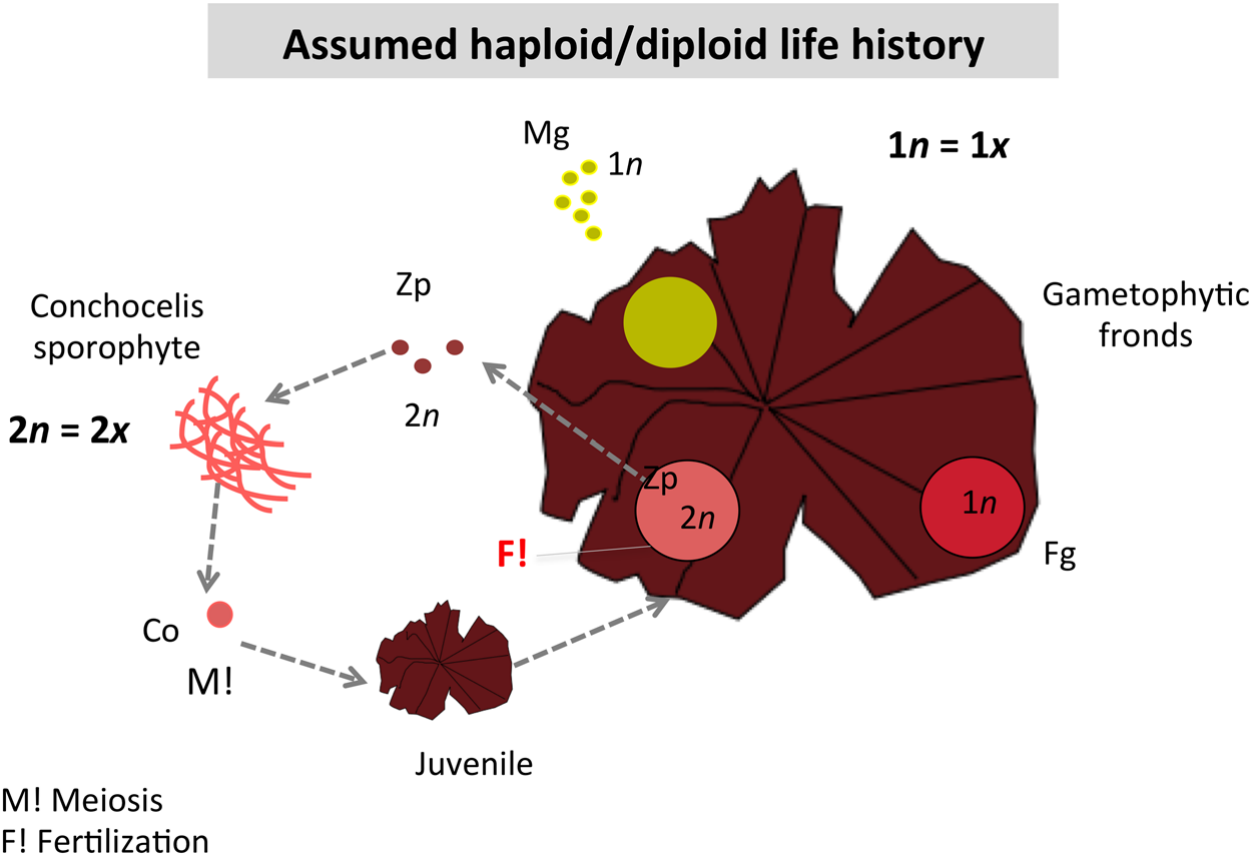
Assumed haploid/diploid life history in *Porphyra* species. M!: Meiosis. F!: Fertilization (syngamy or fusion of males and females gametes). Zp: zygotospores, Mg: Male gametes or Spermatia; Fg: Female gametes; Co: Conchospores.

**Figure 7:**
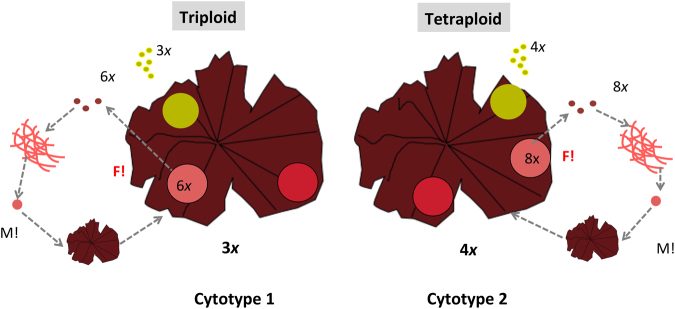
Proposed life history 1 for *Porphyra* species. In life history 1, two genetically distinct lineages coexist, triploids and tetraploids, but they are isolated from each other. M!: Meiosis. F!: Fertilization (syngamy or fusion of males and females gametes).

**Figure 8:**
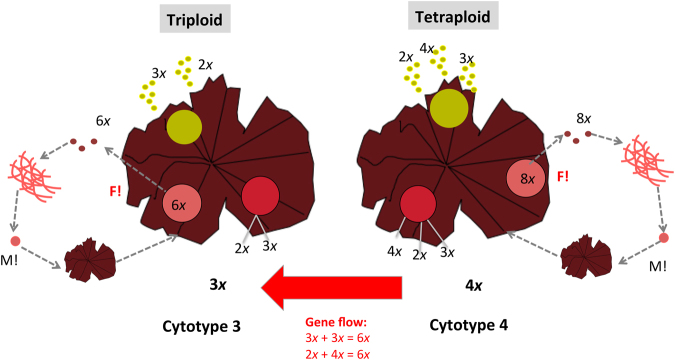
Proposed life history 2 for *Porphyra* species. In life history 2, two genetically distinct lineages coexist, triploids and tetraploids, but they release gametes of different ploidy levels. M!: Meiosis. F! Fertilization (syngamy or fusion of males and females gametes).

**Figure 9:**
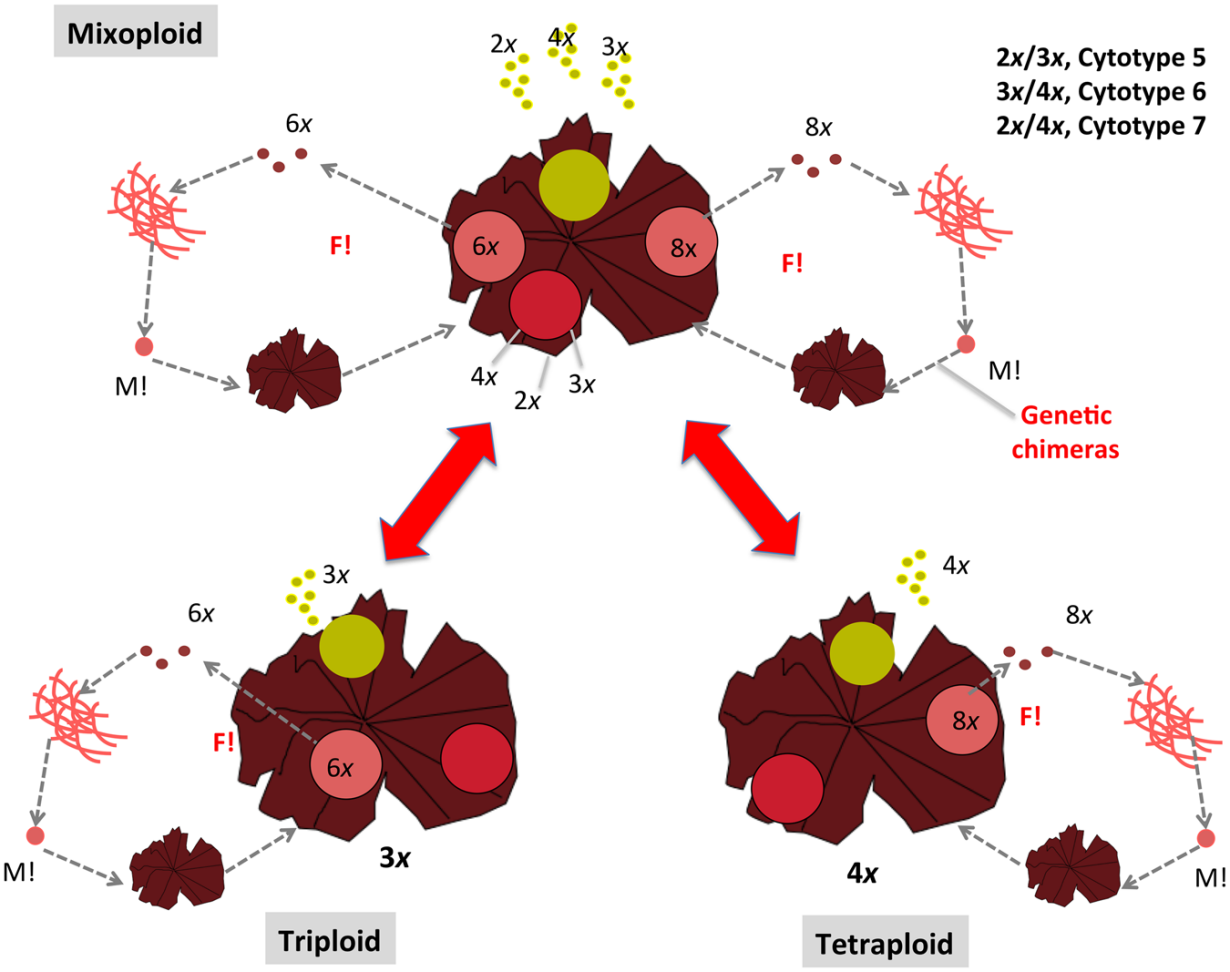
Proposed Life history 3 for *Porphyra* species. In life history 3, three lineages coexist, triploids, tetraploids and mixoploids, with the last lineage acting as bridge between the other two lineages. M!: Meiosis. F!: Fertilization (syngamy or fusion of males and females gametes).

Based on our results, three new life histories are proposed for *Porphyra* species in relation to the general assumption of a haploid/diploid life history (Fig. 6). In life history 1 (Fig. 7), two genetically distinct lineages coexist, triploids and tetraploids, but they are isolated from each other. In life history 2 (Fig. 8), both lineages start to release gametes of different ploidy level. In this way, they can potentially cross with each other. However, since zygotospores 6*x* are only found in triploids, it is believed that gene flow goes from the tetraploids to the triploids. In life history 3 (Fig. 9), three lineages coexist, triploids, tetraploids and mixoploids, with the last lineage the acting as bridge between the other two lineages. Life history 1 and 2 are present in the three *Porphyra* species (*P*. *linearis*, *P*. *umbilicalis* and *P*. *dioica*), while life history 3 is typical of *P*. *linearis*, and present in *P*. *umbilicalis*.

The alternation of generations in algae and land plants typically describe the gametophytic generation as being haploid, and the sporophyte generation as diploid. However, there are many examples, which are not correlated with this haploid/diploid model. For example, the nuclei of sporophytes and gametophytes of the brown seaweed *Haplospora globosa* (Tilopteridales) possess the same number of chromosomes. However, the DNA level of sporophytic nuclei is twice that of gametophytic nuclei^60^. Another example is the gametophyte of Mediterranean *Caulerpas*, which can be diploid or triploid^61^. In *Porphyra*, incongruences in chromosome numbers have also been reported. For example, for some species of *Porphyra*, there is no change in chromosome number between the gametophyte vs. the sporophyte (e.g. refs^62–65^). However, if those species are exhibiting a parthenogenic life history, there is no explanation for the production and release of male gametes unless they were released to fecundate other polyploid races. We wonder now if the incongruences of the life histories reported in the literature for *Porphyra* spp. and other algal/plant taxa may be related to the recurrent presence of polyploid events and/or to the occurrence of mixoploidy undiscovered. And in that case, we believe that many algal groups have to be revisited and the life histories re-described according to the ploidy levels. Knowing that polyploids form at relatively high frequency (1 per 100,000) in flowering plants^10^ and also that formation of polyploids is possible in animals like fish, reptiles, amphibians and even mammals^12,66^, provides support to the hypothesis that many other polyploid algal species might remain undiscovered.

Our results suggest the hypothesis that the debate created on the cryptic diversity in *Porphyra* species may be associated with the diversity of ploidy levels that we found. Since cryptic diversity in *Porphyra* has never been explored in this way, and also considering the evidence for genetic differentiation and isolation for the two polyploid races in *Porphyra linearis*, triploids and tetraploids, we believe they represent distinct evolutionary lineages with incipient speciation in *P*. *linearis* from the Iberian Peninsula, and therefore they may be considered distinct species. Future studies regarding the formal nomenclatural change in *P*. *linearis* are required.

### *Porphyra* as an interesting system to study polyploid evolution

The results of this study highlight the interest in using the genus *Porphyra* to study polyploid evolution. Since the gametophyte is the dominant phase and different cytotypes (triploids, tetraploids and mixoploids and may be other cytotypes) coexist in the same population, the genus *Porphyra* can add novel insight into the evolutionary origins of polyploids. Interactions among these cytotypes can be studied in the same geographical region and hypotheses about intermediate cytotypes that may act as bridges can be formulated and assessed. The sister genus *Bangia* being the oldest eukaryotic multicellular taxon with sexual reproduction recorded^67^, also supports the interest of using the Bangiales to study other evolutionary questions unresolved such as, in an evolutionary scale, what was the first to occur: the gametophytes or sporophyte, and also may help to contrast hypotheses on the origin of the sporophyte and of the alternating generations in land plants^68^. Polyploidy is often associated to asexual reproduction, but in this case all the species studied are sexual, which is also interesting. Besides, many *Porphyra*/*Pyropia* species present asexual reproduction.

Genome doubling (polyploidy) has frequently been associated with evolution and diversification in plants and has been a major factor in the evolution of other lineages of eukaryotes, including yeast^69^ and many groups of vertebrates and invertebrates reviewed in refs^3,70–73^. Here we have reported a polyploid system with a high variability in ploidy levels, cytotypes and lineages with different life history strategies. *Porphyra* is a very interesting genus to continue the study of polyploid evolution, and towards the elaboration of evolutionary models for other red algae, and may be for other eukaryotes.

### Concluding remarks

In our study, many distinct ploidy levels (seven) and genome sizes (eight) were found in *Porphyra* species. These represent two cell lines and comprise seven different cytotype combinations (with single and compound ploidy levels) among the same and different individuals. It is concluded that the gametophytic phase (*n*) in *Porphyra* is not haploid and more than one ploidy level is involved in this phase of the life history. In addition, three main distinct lineages for each *Porphyra* species are found: Triploids, Tetraploids and Mixoploids. In *P*. *linearis*, genetic differentiation was found among the three lineages: triploid and tetraploid, and mixoploid, representing different evolutionary units. Overall, the results of the present study reveal that *Porphyra* spp. constitute a complex polyploid system, composed by autopolyploids, mixoploids, multiploid gametes and probably spontaneous chromosome doubling, where three different types of life histories coexist but in different proportions depending on the species.

## Electronic supplementary material

TABLE S1

DATASET 1

DATASET 2
