# Supplementary material for: Polyploid lineages in the genus Porphyra
Source: Sci Rep. 2018 Jun 6;8:8696. doi: 10.1038/s41598-018-26796-5 (PMC5989262; doi:10.1038/s41598-018-26796-5)
Supplement: Supplementary file 1 — TABLE S1 [file 41598_2018_26796_MOESM1_ESM.pdf]

ELECTRONIC SUPPLEMENTARY MATERIALS

The following information accompanies the article:

Polyploid lineages in the genus *Porphyra*

Elena Varela-Álvarez[1(*)], João Loureiro[2], Cristina Paulino[1] and Ester A. Serrão[1]

[1]CCMAR Centro de Ciências do Mar, CIMAR Laboratório Associado, Universidade do Algarve, Campus de Gambelas, 8005-139 Faro, Portugal.

[2]Centre for Functional Ecology, Department of Life Sciences, University of Coimbra, Calçada Martim de Freitas, 3000-465 Coimbra, Portugal.

(*) Corresponding author: evarela@ualg.pt; elena_varela@yahoo.co.uk

TABLE S1: PCR conditions of 11 microsatellite markers used in this study: Locus name, pcr conditions (T^a (C): annealing temperature/ PCR program: P1, P2, P3 / $MgCl_2$ (mM): Magnesium concentration), and source (For details on the PCR program: P1, P2 and P3, see references)

LOCUS NAME/	T^a (C) /PCR/ $MgCl_2$ (mM)	Source
PoLi_002	58 / P1 / 2	Varela-Álvarez *et al.* 2017a
PoLi_004	56 / P3 / 1.8	Varela-Álvarez *et al.* 2017a
PoLi_005	58 / P1 / 3	Varela-Álvarez *et al.* 2017a
PoLi_006	58 / P1 / 2.5	Varela-Álvarez *et al.* 2017a
PoLi_008	56 / P2 / 2	Varela-Álvarez *et al.* 2017a
PoLi_011	58 / P1 / 2.5	Varela-Álvarez *et al.* 2017a
PoLi_012	58 / P1 / 2.5	Varela-Álvarez *et al.* 2017a
PoLi_015	58 / P1 / 2	Varela-Álvarez *et al.* 2017a
PoLi_017	58 / P1 / 2.5	Varela-Álvarez *et al.* 2017a
PoLi_031	58 / P2 / 2.5	Varela-Álvarez *et al.* 2017a
PoUm_012	56 / P1 / 2	Varela-Álvarez *et al.* 2017b

Varela-Álvarez, E., Paulino, C. & Serrão, E.A. Development and characterization of twelve microsatellite markers for *Porphyra linearis* Greville. *Genetica* **145**: 127-130. (2017)a. doi:10.1007/s10709-016-9941-y.

Varela-Álvarez, E., Balau, A., Paulino, C., Berecibar, E., Pearson, G., & Serrão, E. Isolation and characterization of microsatellite markers for the red alga *Porphyra umbilicalis*. *Plant Genetic Resources,* 1-4. (2017)b. doi:10.1017/S147926211700034X.
